# GIPC1 regulates MACC1-driven metastasis

**DOI:** 10.3389/fonc.2023.1280977

**Published:** 2023-12-08

**Authors:** Franziska Siegel, Hannes Schmidt, Manisha Juneja, Janice Smith, Pia Herrmann, Dennis Kobelt, Kamal Sharma, Iduna Fichtner, Wolfgang Walther, Gunnar Dittmar, Rudolf Volkmer, Fritz G. Rathjen, Peter M. Schlag, Ulrike Stein

**Affiliations:** ^1^ Department Translational Oncology of Solid Tumors, Experimental and Clinical Research Institute, Charité Universitätsmedizin Berlin, and Max-Delbrück-Center for Molecular Medicine, Berlin, Germany; ^2^ Department Developmental Neurobiology, Max-Delbrück-Center for Molecular Medicine, Berlin, Germany; ^3^ German Cancer Consortium, Heidelberg, Germany; ^4^ Experimental Pharmacology and Oncology, GmbH, Berlin, Germany; ^5^ Department Mass Spectrometry, Max-Delbrück-Center for Molecular Medicine, Berlin, Germany; ^6^ Institute for Medicinal Immunology, Charité Universitätsmedizin Berlin, Berlin, Germany; ^7^ Charité Comprehensive Cancer Center, Berlin, Germany

**Keywords:** MACC1, GIPC1, protein-protein interaction, transcription factor, patient survival prognosis

## Abstract

**Background:**

Identification of cancer metastasis-relevant molecular networks is desired to provide the basis for understanding and developing intervention strategies. Here we address the role of GIPC1 in the process of MACC1-driven metastasis. MACC1 is a prognostic indicator for patient metastasis formation and metastasis-free survival. MACC1 controls gene transcription, promotes motility, invasion and proliferation of colon cancer cells *in vitro*, and causes tumor growth and metastasis in mice.

**Methods:**

By using yeast-two-hybrid assay, mass spectrometry, co-immunoprecipitation and peptide array we analyzed GIPC1 protein binding partners, by using the MACC1 gene promoter and chromatin immunoprecipitation and electrophoretic mobility shift assay we probed for GIPC1 as transcription factor. We employed GIPC1/MACC1-manipulated cell lines for *in vitro* and *in vivo* analyses, and we probed the GIPC1/MACC1 impact using human primary colorectal cancer (CRC) tissue.

**Results:**

We identified MACC1 and its paralogue SH3BP4 as protein binding partners of the protein GIPC1, and we also demonstrated the binding of GIPC1 as transcription factor to the MACC1 promoter (TSS to -60 bp). GIPC1 knockdown reduced endogenous, but not CMV promoter-driven MACC1 expression, and diminished MACC1-induced cell migration and invasion. GIPC1 suppression reduced tumor growth and metastasis in mice intrasplenically transplanted with MACC1-overexpressing CRC cells. In human primary CRC specimens, GIPC1 correlates with MACC1 expression and is of prognostic value for metastasis formation and metastasis-free survival. Combination of MACC1 and GIPC1 expression improved patient survival prognosis, whereas SH3BP4 expression did not show any prognostic value.

**Conclusions:**

We identified an important, dual function of GIPC1 - as protein interaction partner and as transcription factor of MACC1 – for tumor progression and cancer metastasis.

## 1 Introduction

Distant metastasis of primary tumors is directly linked to patient survival in many tumor entities. In colorectal cancer (CRC), metastasis accounts for about 90% of patient deaths. CRC is one of the most frequent malignancies in the Western world with more than 1 million of new cases every year. Still, it is one of the leading causes of cancer related deaths (1). The life time risk to suffer from CRC is about 5% in developed countries. Although about half of the subjects with CRC can be cured employing surgery and multimodal treatment, therapy options are limited in particular for patients with metastatic disease. This is reflected by 5-year-survival rates higher than 90% for patients in early stages, which drop to 65% in patients initially diagnosed with regional lymph node metastases and is further reduced to less than 10% in patients with distant metastases. About 30% of CRC patients are diagnosed with distant metastases, and despite follow-up curative primary treatments, at least a further third will develop metastases later. Thus, the formation of distant metastases is the decisive and most lethal event during the course of the disease. It critically limits successful therapy and is the most frequent cause of treatment failure (2).

Here, we focus on the cancer- and metastasis-inducing gene Metastasis-associated in colon cancer 1 (MACC1). MACC1, first identified as novel gene in tissues of colon cancer patients (3, 4), is a predictor for CRC metastasis and metastasis-free survival. MACC1 levels in the primary tumors or patient blood were significantly higher in cancers that metachronously developed distant metastases compared to those which did not metastasize (3, 5). The 5-year-survival rate was 80% for patients with low MACC1, compared to 15% for patients with high MACC1 expression in their primary tumors.

MACC1 induces phenotypes such as proliferation, migration, invasion, dissemination, alters apoptosis (6), inflammation (7), drug resistance (8), biomechanics (9), protein trafficking (10), metabolisms (11) and platelet communication (12). Further, MACC1 induced tumorigenesis and metastases in several mouse models (3, 13, 14). We and many other groups have shown the clinical importance of MACC1 as causal, prognostic and predictive biomarker for more than 20 solid cancer types, including meta-analyses for solid cancers, cancers of the digestive system, colorectal, hepatocellular, gastric, gynecological and breast cancer (reviewed in 4). MACC1 shows 49.3/43.7% (nt/aa) sequence identity to only one known human homologue gene: SH3BP4 (SH3-domain binding protein 4) (15, 16). SH3BP4 is a ubiquitously expressed 107.5 kDa intracellular protein suggested to function in cargo-specific control of clathrin endocytosis (17). In contrast to MACC1, SH3BP4 did neither induce cell migration nor invasion in colon cancer cells.

The ubiquitously expressed 36 kDa protein GIPC1 (GAIP C-Terminus-Interacting Protein PDZ Domain Containing Family, Member 1), also described as Synectin, TIP-2 or GLUT1CBP, was initially identified as binding partner of the GTPase-activating protein RGS-GAIP (regulator of G-protein signaling-GTPase activating protein for Gαi) (18). It belongs to the GIPC protein family consisting of GIPC1, 2 and 3 (19). The GIPC1 protein contains a central PDZ (PSD-95, discs large, zona occludens 1) domain that binds type 1 PDZ-binding motifs in a variety of proteins. These proteins include adhesion molecules and transmembrane receptors like Integrin α5 and α6A/B subunits, receptor tyrosine kinases TrkA, TrkB and IGF-1R, the Frizzled 3 class of Wnt receptor and TGF-ß type III receptor (reviewed in 20). GIPC1 has been suggested to be involved in cancer development, progression, and metastasis (20–26). It plays multiple roles regulating signaling pathways in a variety of tumor types leading to altered protein trafficking and endocytosis, to increased cell proliferation and tumor growth, angiogenesis, migration, invasion and metastasis via IGF1/IGF-1R, Akt-Mdm2-p53, MMP9-Cdc42, VEGFA or integrins (20, 26–33).

Here we address the role of GIPC1 in the context of the MACC1 network thereby identifying GIPC1 as being important for MACC1-induced metastasis formation in CRC. We tested for protein-protein interactions of MACC1 and GIPC1 and established their specific interaction sites. We proved for binding of GIPC1 to the core promoter of MACC1 and determined its relevance as transcription factor for MACC1. We validated these newly identified dual functions of the novel actor GIPC1 in the MACC1 context by functional assays *in vitro* and *in vivo*. Finally, we tested our hypothesis for its importance as prognostic biomarker in the context of MACC1 for survival of CRC patients.

## 2 Material and methods

All information concerning materials and methods - cell lines, cell clones, RNAi; plasmid constructs; generation of MACC1 promoter constructs; RNA isolation and quantitative PCR; luciferase promoter activity assay; chromatin immunoprecipitation assay (ChIP); nuclear extract preparation and electrophoretic mobility shift assay (EMSA); protein extraction, Western blotting, co-immunoprecipitation; subcellular fractionation; yeast two-hybrid (Y2H) analysis; mass spectrometry; pepspot analysis; xenografting and *in vivo* experiments; patients and tissues and statistics - can be found in the **Supplementary Methods** part.

Ethical agreement for *in vivo* experiments: The welfare of the animals was maintained in accordance with the general principles governing the use of animals in experiments of the European Communities and German legislation. The study was performed in accordance to the United Kingdom Coordinating Committee on Cancer Research (UKCCCR) regulations for the Welfare of Animals and of the German Animal Protection Law and approved by the local responsible authorities, Berlin, Germany (State Office of Health and Social Affairs, Berlin, Germany), approval No. G 0333/18).

Ethics approval and patient consent to participate: All analyses were carried out in accordance with the guidelines approved by the institutional review board, number AA3/03/45, of the Charité—Universitätsmedizin Berlin, Germany. All patients gave written informed consent and the authors complied with all relevant ethical regulations for research involving human participants.

## 3 Results

### 3.1 GIPC1 is a newly identified protein binding partner of the metastasis inducer MACC1

#### 3.1.1 MACC1 interacts with GIPC1

**Figure 1:**
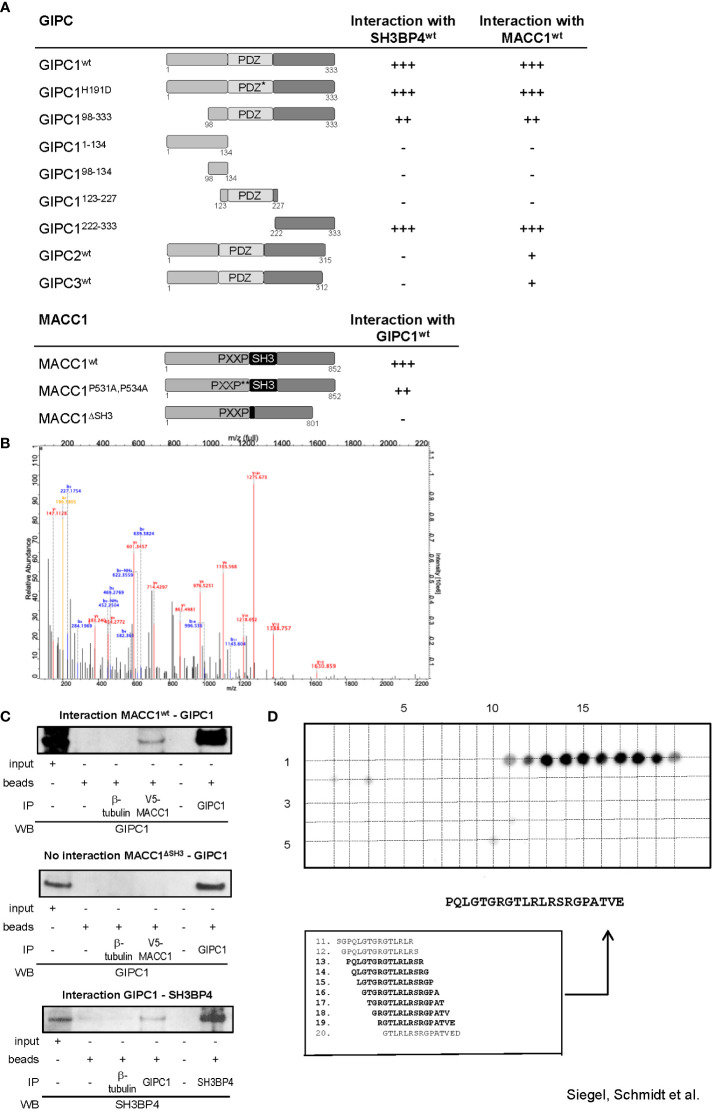
GIPC1 is a newly identified protein binding partner of the metastasis inducer protein MACC1. **(A)** Physical interaction of GIPC1 and different mutant polypeptides thereof were analyzed for binding to MACC1 and to SH3BP4 by Y2H in Y187 yeast cells. The C-terminal region of GIPC1 (aa 222-333) was identified to be essential for these interactions. MACC1 and mutants thereof were analyzed for binding to GIPC1 by Y2H. The SH3-domain of MACC1 (aa 552-618) was identified to be essential for this interaction. The original results of the b-galactosidase staining of transformed yeast colonies are shown in **Supplementary Figure 1**. **(B)** Tandem MS spectrum for GIPC peptide obtained from cLC-ESI-MS analysis. The sequence of this peptide was verified by the presence of complementary B ions (NH2-terminus–derived fragment ions) and Y ions (COOH-terminus–derived fragment ions). We performed two independent IPs of MACC1 from SW620 cells with high endogenous MACC1 expression employing two different MACC1 antibodies (four experiments), which were subjected to shot-gun MS. In total, 1203 proteins were identified, **(C)** Validation of the physical interaction of GIPC1 to MACC1 and of GIPC1 to SH3BP4 by co-immunoprecipitation in SW480 cells. No interaction with GIPC1 was found when using MACC1 mutants lacking the SH3 domain confirming the co-immunoprecipitation data (input: 2% of total protein). **(D)** Pepspot analysis. The peptide array displays 98 peptides in 5 rows and 20 columns. The peptides represent overlapping 15mer-sequences of the GIPC1 C-terminus (222-333). The array was probed for binding MACC1. Spots 13-19 show sufficient spot signal intensity and were selectively recognized by MACC1. Spot-sequence correlation and deducing the GIPC1 binding site for MACC1.

We started our analyses by yeast two-hybrid binding studies. For this purpose, the full-length cDNA sequences of MACC1 and GIPC1 as well as of several mutant variants were cloned into the Gal4 DNA binding domain vector pGBT9 and the GAL4 DNA activation domain vectors pGAD10 or pGAD424. A β-galactosidase filter assay of the yeast reporter strain Y187 co-transformed with appropriate combinations of the fusion constructs confirmed the interaction between MACC1 and GIPC1 (**Figure 1A**). Interestingly, SH3BP4 - a homologue of MACC1 implicated in cargo-specific control of clathrin-mediated endocytosis (15–17) - was also found to interact with GIPC1. The central Post synaptic density protein (PSD95), Drosophila disc large tumor suppressor (Dlg1), and Zonula occludens-1 protein (zo-1) (PDZ) domain of GIPC1 has been shown to interact with several transmembrane proteins (26).

To investigate whether the association of MACC1 and GIPC1 occurs through the PDZ domain we generated a mutant variant of GIPC1 by site directed mutagenesis in which the function of the PDZ binding pocket is compromised by the replacement of the histidine residue 191 at position 1 of the α helix B of the PDZ domain by aspartic acid (H191D). This point mutation resulted in a complete loss of the previously reported interaction between GIPC1 and neuropilin1 (34). However, the interaction between MACC1 and GIPC1 was not affected by the mutated PDZ domain (**Figure 1A**). In contrast, the use of truncated versions of GIPC1 comprising its C-terminal, central PDZ or N-terminal segments demonstrated that the 111 C-terminal amino acid residues of GIPC1 are sufficient for an interaction with MACC1 (**Figure 1A**). Yeast two-hybrid binding studies analyzing the interaction of MACC1 with the other members of the GIPC-family showed a weaker binding of GIPC2 and GIPC3 to MACC1. Rat SH3BP4 was found to also strongly interact with mouse GIPC2 but not human GIPC2 or -3.

To identify segments of the MACC1 protein that are involved in the interaction with GIPC1 we tested two mutant variants of MACC1: a double point mutant in which the two proline residues of the PXXP-motif were converted to alanine (P531A, P534A) and a deletion mutant lacking the SH3 domain (ΔSH3) (3). Whereas interaction was preserved in MACC1^P531A,P534A^ the deletion of the SH3 domain of MACC1 resulted in a complete loss of interaction (**Figure 1A**). In summary, analysis of mutant constructs demonstrated that the C-terminal segment of GIPC1, region aa 222-333, is essential for binding to MACC1 and its relative SH3BP4 while the SH3 domain of MACC1 (aa 552-618) is involved in this interaction. The original results of the β-galactosidase staining of transformed yeast colonies are shown in **Supplementary Figure 1**.

#### 3.1.2 Mass spectrometry confirms interaction of GIPC1 with MACC1

To verify the interaction between MACC1 and GIPC1 as detected by yeast two-hybrid binding studies we performed mass spectrometry analysis measuring the MACC1 interactome by quantitative proteomics in SW620 cells. First, we characterized the specificity of the antibodies intended for immunoprecipitation by mass spectrometry. Both antibodies we employed for pulling-down MACC1 were confirmed by shot-gun mass spectrometry. We performed two independent MACC1 immunoprecipitations from SW620 cells with high endogenous MACC1 and subjected the eluates to quantitative proteomic analysis. In total 1203 proteins were identified, among them we also found GIPC1 as a putative interaction partner of MACC1 (GSE70458). We analyzed the MACC1 interactome for enrichment of GO terms linked to cellular response to extrinsic factors and associated signal transduction pathways (35). The tandem mass spectrometry spectrum for the GIPC1 peptide was obtained from cLC-ESI-MS analysis. The sequence of this peptide was verified by the presence of complementary B ions (NH2-terminus–derived fragment ions) and Y ions (COOH-terminus–derived fragment ions) (**Figure 1B**). Taken together, mass spectrometry confirmed the interaction of MACC1 and GIPC1 found by yeast two-hybrid binding studies.

#### 3.1.3 Co-immunoprecipitation confirms interaction of GIPC1 with wildtype MACC1

Independent confirmation of the mass spectrometry and yeast two-hybrid physical interaction data of MACC1 and GIPC1 was obtained by immunoprecipitation using SW480/MACC1 cells ectopically overexpressing V5-tagged MACC1. Lysates were immunoprecipitated with IgG, β-tubulin (negative controls), V5-MACC1 and GIPC1 antibodies, and Western Blot was performed with GIPC1 antibody for wildtype MACC1- or MACC1ΔSH3-GIPC1 interaction, or with SH3BP4 antibody for GIPC1-SH3BP4 interaction. Thereby, we validated the interaction of GIPC1 with wildtype MACC1 as well as of GIPC1 with SH3BP4 (**Figure 1C**). No interaction was found for GIPC1 and the MACC1 mutant lacking the SH3 domain in SW480/ΔSH3 cells, expressing ectopically V5-tagged ΔSH3-MACC1 (**Figure 1C**).

#### 3.1.4 The linear sequence motif PQLGTGRGTLRLRSRGPATVE of GIPC1 binds to MACC1

Next, the binding site for MACC1 at the protein GIPC1 was determined performing binding assays on cellulose membrane-bound peptide arrays. A SPOT-synthesized peptide array displaying a set of overlapping peptides spanning the C-terminal part of GIPC1 (sequence position 222-333) was probed for binding Flag-tagged MACC1. The spots 11-20 were selectively recognized by MACC1 whereupon spots 13-19 were analyzed of sufficient signal intensity (**Figure 1D**). As a result, the linear sequence motif PQLGTGRGTLRLRSRGPATVE of GIPC1 (position 234-254) is deduced as the binding site for MACC1, with the GIPC1 region 241-247 aa for being most responsible for the physical interaction with MACC1 (**Figure 1D**).

Taken together, we demonstrate with different technologies a physical protein-protein binding of GIPC1 to MACC1 and confined the binding regions to aa 241-247 for GIPC1 and to aa 552-618 for MACC1.

### 3.2 GIPC1 is a newly identified transcription factor and binds to the MACC1 gene promoter

#### 3.2.1 Subcellular localization of GIPC1

**Figure 2:**
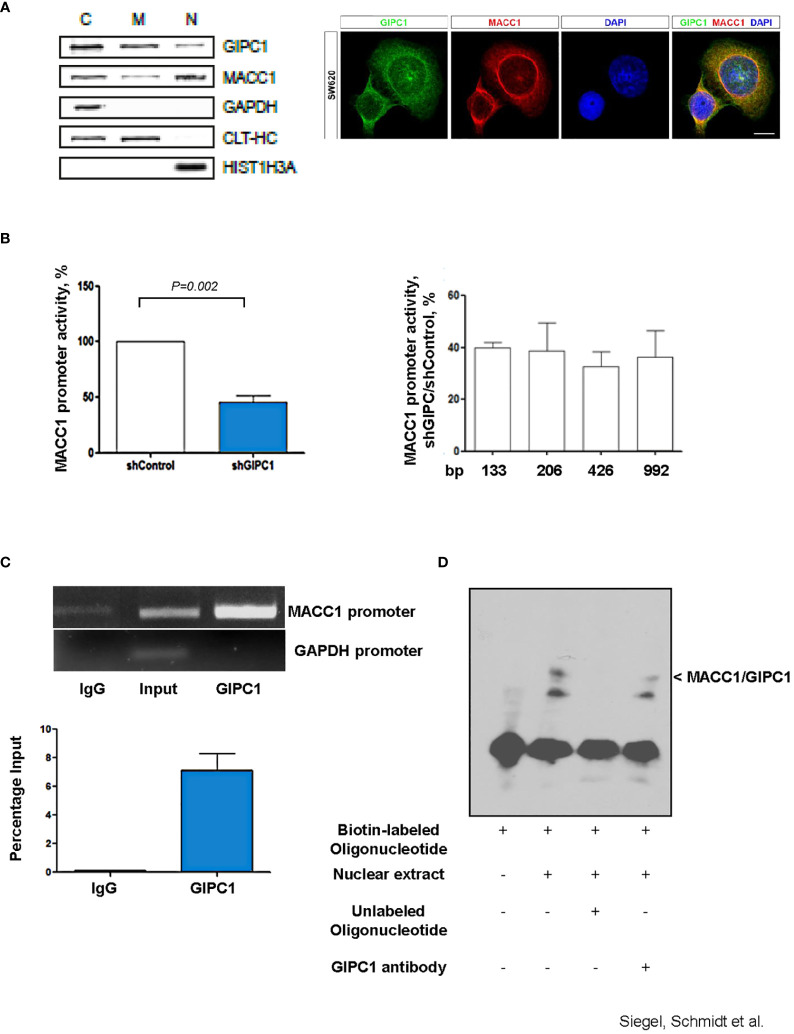
GIPC1 is a newly identified transcription factor and binds to the MACC1 gene promoter. **(A)** Left: Subcellular distribution of GIPC1 and MACC1 in the cytoplasm (C), the membranous fraction (M) and the nucleus (N) of SW620 cells. GAPDH (cytoplasm), CLTC (cytoplasm and membranous fraction) and HIST1H3A (nucleus) served as controls. Interestingly, GIPC1 and MACC1 were both found also in the nucleus. Right: By immunofluorescence, the subcellular localization of GIPC1 and MACC1 was validated, predominantly the presence of both proteins in the nuclei. These data are in harmony with those from in silico predictions (https://www.genecards.org/cgi-bin/carddisp.pl?gene=GIPC1). The intended co-staining for GIPC1 and MACC1 in SW620 cells is shown with GIPC1 in green, MACC1 in red and DAPI in blue. Scale bar 10 mm. **(B)** Left: Impact of GIPC1 on the MACC1 promoter activity. MACC1 promoter luciferase reporter construct was co-transfected along with pGL4.74 Renilla plasmid in SW620-shcontrol and SW620-shGIPC1 cells. After 24 h of transfection, luciferase activity was measured and was normalized to Renilla luciferase activity to correct for the variation in transfection efficiencies. Luciferase activity from the SW620-shcontrol cells was set to 100%. shGIPC1-transfected cells showed a significantly reduced MACC1 promoter activity. Right: Fragments of the MACC1 promoter with deletions at the 5’ end and possessing a common 3’-end were inserted into pGL4.17 and were transfected into SW620-shGIPC1 and SW620-shControl cells. Luciferase activity was normalized to Renilla values and expressed as percentage reduction of luciferase activity in SW620-shGIPC1 as compared with SW620-shcontrol cells. The MACC1 promoter fragment -18 to -133 bp is sufficient to demonstrate MACC1 promoter activity loss, which is not further enhanced by larger deletions. **(C)** ChIP assay. SW620 chromatin was immunoprecipitated with a GIPC1 antibody and quantified by agarose gel electrophoresis, using a primer set specific for the MACC1 promoter and GAPDH. Non-immune IgG and input DNA without any immunoprecipitation with antibody served as negative and positive controls respectively. The binding of GIPC1 to the MACC1 promoter is demonstrated. **(D)** EMSA. 5’-end biotin labeled oligonucleotide corresponding to human MACC1 promoter were incubated alone as well as with nuclear extracts from SW620 cells along with 100x molar excess of unlabeled competitor sequence or with the antibody specific for GIPC1 to indicate specificity<city/> of the protein-DNA complexes. The reactions were analyzed by polyacrylamide gel electrophoresis. GIPC1 binds to the first 60 bp of the MACC1 promoter.

We performed subcellular fractionation of SW620 CRC cells and detected GIPC1 in the cytoplasmic, membranous as well as in the nuclear fractions (**Figure 2A**). Similarly, MACC1, was detected in the cytoplasmic and in the nuclear fraction as previously reported (3). Immunofluorescent co-staining of GIPC1 and MACC1 in SW620 shows a partial overlap in the distribution of both proteins in the cytosol as well as their presence in the nuclei (**Figure 2A**). Consistent to our finding, a nucleic localization of GIPC1 has been previously reported and is also found and published via https://www.genecards.org/cgi-bin/carddisp.pl?gene=GIPC1.

#### 3.2.2 GIPC1 regulates the MACC1 gene promoter activity

Since silencing GIPC1 is already an acknowledged tool in cancer research (31), and GIPC1 was found in the nuclei, we tested for a possible action of GIPC1 on the regulation of MACC1 gene expression. Previously, we were successful to identify a 5’-upstream region of the MACC1 gene that demonstrates gene promoter activity. This finding enables us to probe for a potential GIPC1 role acting as transcription factor on MACC1 (36).

We transfected SW620/shControl and SW620/shGIPC1 cells with the MACC1 promoter luciferase construct MACC1p-992 (36) or empty vector. 24 h after transfection, we measured the luciferase activity. We found a 48% decrease in the MACC1 activity in GIPC1 knock down cells (P=0.02; **Figure 2B**) indicating the possible interaction of GIPC1 with the MACC1 gene promoter. We were inquisitive about the region where it binds on the MACC1 promoter. Thus, we transfected the 5’-truncated MACC1 promoter constructs MACC1p-426, MACC1p-206, and MACC1p-133 (36) in SW620/shControl and SW620/shGIPC1 cells and calculated the change in the MACC1 promoter activity. We detected no significant variations in the MACC1 promoter activity among the different promoter constructs (**Figure 2B**) suggesting that the smallest MACC1 promoter fragment MACC1p-133 is sufficient for the MACC1-GIPC1 interaction.

#### 3.2.3 GIPC1 physically interacts with the MACC1 gene promoter

We then tested for a potential physical binding of GIPC1 to the MACC1 promoter by chromatin immunoprecipitation (ChIP) and by electrophoretic mobility shift assay (EMSA), thereby analyzing the GIPC1 binding to the authentic and artificial MACC1 gene promoter respectively (**Figures 2C, D**). First, we analyzed the physical interaction of GIPC1 with the MACC1 promoter in SW620 cells by ChIP assay. SW620 chromatin was immunoprecipitated with a GIPC1 antibody, reactions with an IgG antibody or loading input DNA served as negative and positive controls, respectively. MACC1 and GAPDH promoter regions were amplified by PCR. A clear strong band was seen on amplification of immune precipitated chromatin by using the GIPC1 antibody with the primers for the MACC1 gene promoter. This demonstrates the binding of the GIPC1 protein to the region –18 bp to -133 bp of the MACC1 promoter. No binding of the GIPC1 protein to a GAPDH sequence was observed, which was used as a control (**Figure 2C**).

For EMSA, we designed biotin labeled oligonucleotides corresponding to the first 60 bp from the transcription start site in the MACC1 promoter. Incubating the 5’-labeled MACC1 promoter fragment with nuclear extract from SW620 cells, we observed a DNA-protein complex formation which disappeared on adding 100fold molar excess of unlabeled promoter fragment. The specificity of the complex was determined by addition of an antibody for GIPC1 which led to a decrease in the intensity of the specific shift (**Figure 2D**).

Taken together, the results from both ChIP and EMSA analysis confirmed the physical binding of GIPC1 to the MACC1 gene promoter, confined to the transcription start site to –60 bp.

### 3.3 GIPC1 regulates MACC1 expression and MACC1-induced cell motility *in vitro*

#### 3.3.1 Correlative expression of GIPC1 and MACC1

**Figure 3:**
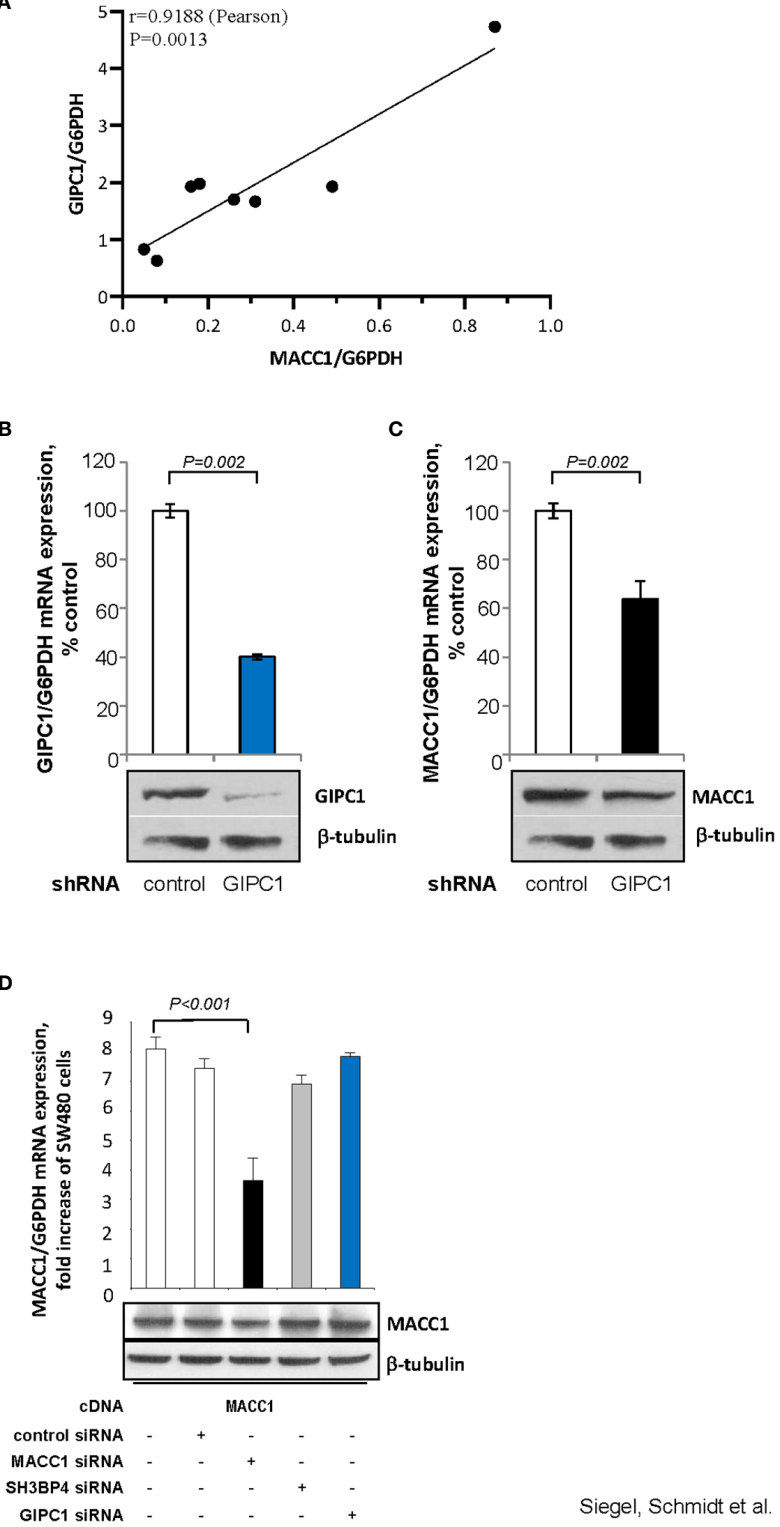
GIPC1 regulates MACC1 expression and MACC1-induced cell motility *in vitro.* **(A)** Correlation of GIPC1 and MACC1 expression levels in different CRC cell lines. GIPC1 and MACC1 expression levels were determined in the same sample of a variety of several CRC cell lines by qRT-PCR, normalized using G6PDH and sorted by ascending MACC1/G6PDH mRNA levels of CRC cell lines (HT29, WIDR, HCT116, HCT15, HCA7, DLD1, Caco-2, SW48). We found a positive statistically significant correlation with a Pearson r of 0.9188 and a P value of 0.0013. **(B, C)** Impact of GIPC1 knock-down in SW620 cells on **(B)** GIPC1 expression and **(C)** on MACC1 expression on mRNA and protein levels (both P=0.002). shRNA transfection was done to knock down GIPC1. Stable clones were isolated and validated. Total RNA was isolated from these cells, reverse transcribed and quantified using real time PCR. The data is normalized for G6PDH. Results are shown as means ± SEM of three independent experiments performed in duplicate. **(D)** Expression of MACC1 in dependence of MACC1, SH3BP4 and GIPC1 knock down in ectopically MACC1-overexpressing cells SW480/MACC1 cells (CMV-promoter-driven). Cells were treated with siRNAs either acting on MACC1, on SH3BP4 or on GIPC1, compared to control siRNA-treated cells. Treatment with MACC1 siRNA resulted in decreased MACC1 expression (P<0.001), whereas siRNA acting on SH3BP4 or GIPC1 did not affect the CMV promoter-driven forced MACC1 expression, neither at RNA nor at protein level.

First, we analyzed a panel of CRC cell lines for mRNA expression of GIPC1 and MACC1 by qRT-PCR, normalized using G6PDH and sorted by ascending MACC1/G6PDH mRNA levels (order: HT29, WIDR, HCT116, HCT15, HCA7, DLD1, Caco-2, SW48). We observed a positive statistically significant correlation with a Pearson r=0.9188 and a P=0.0013 (**Figure 3A**).

#### 3.3.2 GIPC1 silencing down regulates MACC1 expression

Next, we aimed to evaluate whether GIPC1 silencing results in altered levels of MACC1 also in SW620 cells that express MACC1 at the highest endogenous level compared to the cell line panel. In SW620/shGIPC1 cells, we measured a knock down of GIPC1 mRNA to 40%, compared to SW620/shControl cells (P=0.002; **Figure 3B**). This GIPC1 knock down resulted also in an about 40% decreased MACC1 mRNA expression (P=0.002; **Figure 3C**). GIPC1 and MACC1 expression reductions following GIPC1 silencing were also observed at the protein levels.

As control, we investigated whether GIPC1 silencing also led to altered levels of MACC1 in SW480 cells ectopically overexpressing MACC1. SW480/MACC1 cells were treated with siRNAs either acting on MACC1 or on GIPC1, compared to siControl-treated cells. Based on the 43.7% aa similarity of MACC1 and its paralogue SH3BP4 and on the validated GIPC1-SH3BP4 protein-protein binding (**Figure 3D**), we also employed siRNA targeting SH3BP4. Treatment with MACC1 siRNA resulted in decreased MACC1 expression (P<0.001), whereas siRNA acting on SH3BP4 or on GIPC1 did not affect MACC1 expression, neither on RNA nor on protein level in these ectopically MACC1 overexpressing cells.

### 3.4 GIPC1 silencing reduces CRC metastasis in mice

**Figure 4:**
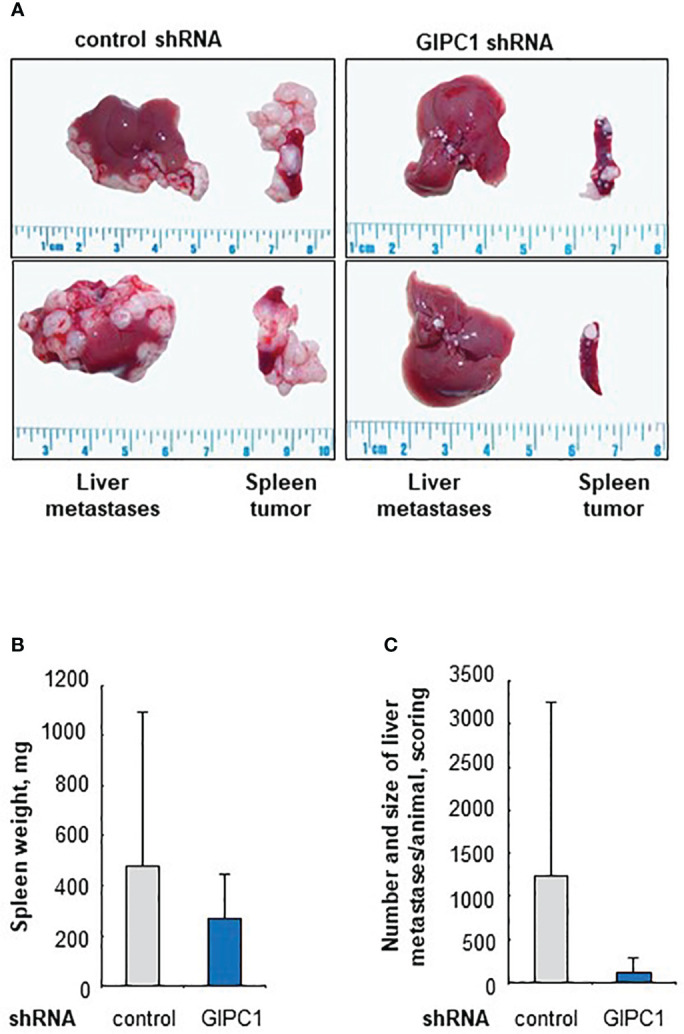
GIPC1 silencing reduces CRC metastasis in mice **(A)** Tumor growth at the spleen and liver metastasis formation of SW620 cells is reduced after GIPC1 knock-down. SW620/shControl cells and SW620/shGIPC1 cells were intraspenically transplanted, Left: Isolated organs, spleens and livers, originating from intrasplenically transplanted primary SW620-tumors and metastasis after treatment with control shRNA, exemplified for two animals. Right: Isolated organs, spleens and livers, with primary SW620-tumors and metastasis after treatment with GIPC1 shRNA, exemplified for two animals. A clear reduction of tumor growth and metastasis formation is observed after GIPC1 shRNA treatment. **(B)** Spleen weight. This is illustrated by clearly reduced weight of spleens inclusive primary tumors of shGIPC1-mice vs. control mice. **(C)** Most importantly, GIPC1 knock-down resulted in reduced number and size of metastases/animal of shGIPC1-mice vs. control mice.

For the following *in vivo* experiments, we used the SW620 cells with high endogenous MACC1 expression treated with and without shGIPC1 RNA in order to prove for the effects of GIPC1 silencing on tumor growth and metastasis formation in xenografted mice. NMRI:nu/nu mice were intrasplenically transplanted with stably transfected SW620/shControl or SW620/shGIPC1 cells. Read outs are tumor growth at the spleen (site of transplantation) and development of metastases at the liver (**Figure 4A**). All mice, either transplanted with SW620/shControl or with SW620/shGIPC1 cells developed a spleen tumor. Mice were sacrificed on day 40 due to ethical reasons, since some mice in the shControl group developed palpable spleen tumors. Spleens and livers were removed and pictures of the organs were taken.

Animals transplanted with SW620/shGIPC1 cells showed a clear, but non-significant reduction in growth of the primary tumor at the spleen when compared to SW620/shControl-transplanted animals, with 0.27 g spleen weight (SD 0.18) compared to 0.48 g (SD 0.67) spleen weight of the control group animals (**Figure 4B**). However, when analyzing the metastasis score considering number and size of the formed liver metastases, we found a 10-fold lower metastasis score in the shGIPC1 group when compared to the shControl mice, with a score of 118 (SD 179; shGIPC1 group) compared to 1225 (SD 2019) in the control group animals (**Figure 4C**). Body weight was not affected.

### 3.5 GIPC1 expression correlates with MACC1 expression and with shorter metastasis-free survival of CRC patients

#### 3.5.1 GIPC1 correlates with shorter metastasis-free survival of CRC patients

We aimed at evaluating GIPC1 alone and also in combination with MACC1, for its potential value of being a prognostic biomarker. Therefore, we determined the mRNA expression levels of GIPC1 in addition to MACC1 in a cohort of 59 non-treated human primary CRC specimens of stages I, II, and III, i.e. not distantly metastasized at the time point of analysis. Sectioned cryo-preserved tissues were evaluated by a pathologist. Tumor cell populations were microdissected, and gene specific qRT-PCR was performed. All patient classifications in low and high expressors were based on receiver operating characteristic (ROC)-calculated cut-offs.

**Figure 5:**
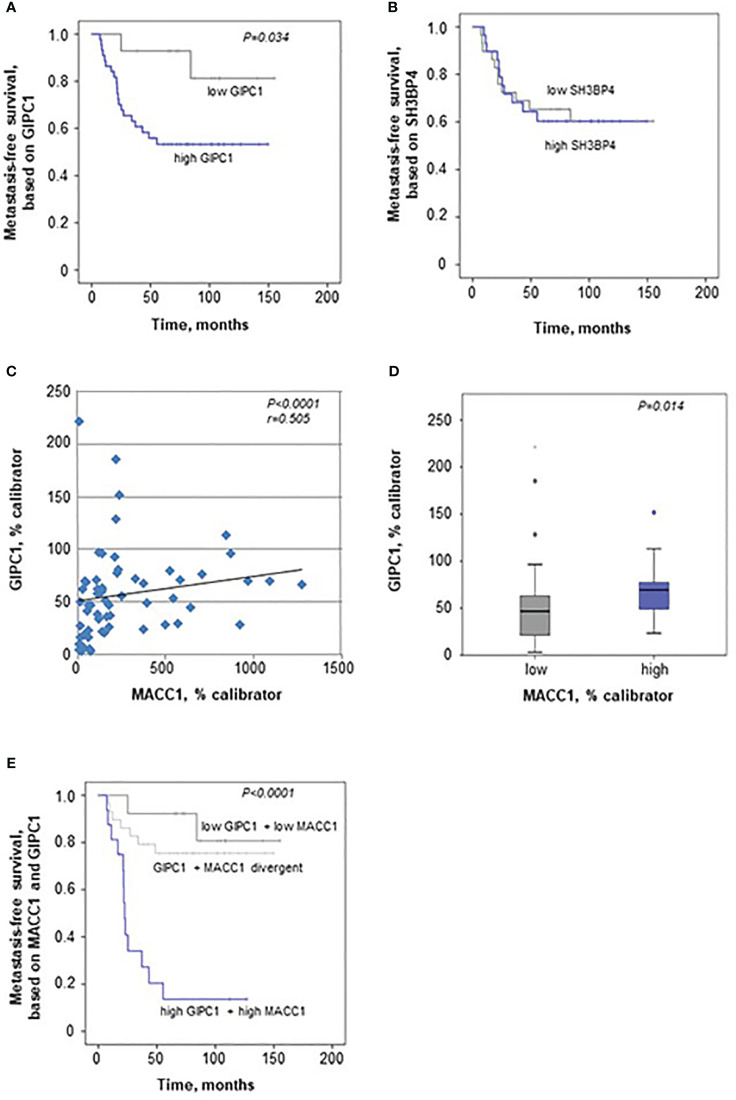
GIPC1 is prognostic for CRC metastasis and improves CRC patient prognosis when combined with MACC1. **(A, B)** Expression of GIPC1 mRNA **(A)** and SH3BP4 **(B)** was determined by quantitative real time RT-PCR with RNA isolated from microdissected tumor cell population of primary, not (yet) metastasized tumors in stages I, II and III (n=59). GIPC1 expression serves as prognostic factor for the formation of distant metastases in CRC patients (P=0.034), with significantly worse metastasis-free survival in the GIPC1 high-expressing patients. SH3BP4, the only relative of MACC1 with a 43.7% of aa sequence identity, was determined simultaneously in the same tumors from the same RNA sample, but did not show any prognostic value. **(C)** For expression of MACC1 in the same patient cohort ((P<0.0001; even same RNA sample) and for patient characteristics: see (3). Analyzing an expression correlation of MACC1 and GIPC1 in the CRC tumors, we found a positive statistically significant correlation with a Pearson r of 0.505 and a P value of <0.0001. **(D)** For correlation analysis of MACC1 expression and GIPC1 expression, tumors were classified due to low and high expressing groups, we found a statistically significant expression for GIPC1 and MACC1 expression (P=0.014). **(E)** Combining high MACC1 and high GIPC1 (qRT-PCR values) vs. low MACC1 and low GIPC1 patients showed improved prognostic values (P<0.0001) when compared to MACC1 expression alone (3) or GIPC1 expression alone **(A)**.

When evaluating GIPC1 as a potential prognostic marker for patient survival, we performed Kaplan-Meier survival analysis based on the GIPC1 expression in the primary tumors, staged I, II, and III. Interestingly, we found that expression of GIPC1 might serve as prognostic factor for CRC. Patients high expressing GIPC1 in their primary tumors (n=44) showed significantly shorter MFS times than GIPC1 low expressors (n=15; P=0.034; **Figure 5A**). GIPC1 high expressing patients had a median MFS of 62.78 months (SD 51.27), whereas GIPC1 low expressors showed a median MFS of 110.27 months (SD 46.14). The median MFS was about 47.5 months shorter in the GIPC1 high expressing patient group. SH3BP4, however, also determined in the primary tumor, is not of prognostic value with respect to MFS (**Figure 5B**).

#### 3.5.2 GIPC1 expression correlates with MACC1 expression values in primary CRC tumors

We also analyzed MACC1 in the same patient samples of the cohort of 59 CRC patients, as previously described (3). MACC1 high expressing patients had a median MFS of 23.8 months (SD 38.74), whereas MACC1 low expressors showed a median MFS of 103.43 months (SD 46.73) (P<0.0001). The median MFS was about 79 months shorter in the MACC1 high expressing patient group.

When correlating the expressions of GIPC1 and MACC1 in all the tumors, we found a moderate correlation expression (Spearman r=0.505, P<0.0001; **Figure 5C**). We did not find any dependence of GIPC1 expression on sex, age (<60 vs. >60 years), or stage (I, II, or III).

When classifying the tumors in a low and a high MACC1 expressing group, GIPC1 expression was higher in those colorectal tumors that also express MACC1 at high levels (median 69.72 GIPC1 mRNA/% calibrator vs. 46.78 GIPC1 mRNA/% calibrator in the low expressing group, P=0.014; **Figure 5D**).

#### 3.5.3 Combining GIPC1 expression and MACC1 expression improve patient prognosis

We also did a combinatorial analysis of GIPC1 expression together with MACC1 expression in order to test if the patient prognosis based on one marker alone, GIPC1 or MACC1, can be improved when both markers were combined (**Figure 5E**). Based on this combination, 3 subcohorts were defined: Patients with GIPC1 high and MACC1 high showed the worst median MFS of 22.6 months (SD 39.66 months, n=17). Patients with divergent GIPC1 and MACC1 expression values (GIPC1 high/MACC1 low, GIPC1 low/MACC1 high) had a median MFS of 102.06 months (SD 46.92, n=28). Patients with GIPC1 low and MACC1 low had the best median MFS of 113.14 months (SD 46.3 months, n=14). Patients expressing both biomarkers high showed significantly different MFS times compared with patients expressing both biomarkers low (P<0.001) and had a 91 months shorter median MFS than patients in the GIPC1 low/MACC1 low group. In the GIPC1 high/MACC1 high patient group, 82.4% of the patients developed metachronously distant metastases (false positive rate: 17.6%); in the GIPC1 low/MACC1 low patient group, 85.7% of the patients did not develop any distant metastases (false negative rate 14.3%). Thus, the combination of GIPC1 with MACC1 improved patient prognosis based on GIPC1 alone and might contribute to better identify those patients who are at high risk.

## 4 Discussion

Here we report the novel, dual impact of GIPC1 in the process of MACC1-driven metastasis. We identified GIPC1 as protein binding partner of the MACC1 protein by mass spectrometry, yeast-two-hybrid assay, co-immunoprecipitation and peptide array: with aa 241-247 of GIPC1 binding to MACC1. We further demonstrated the binding of GIPC1 as transcription factor to the MACC1 promoter by ChIP and EMSA at position from transcription start site to -60 bp). Knockdown of GIPC1 reduced endogenous, but not CMV promoter-driven MACC1 expression, thereby diminishing MACC1-induced cell migration and invasion, which underlines the role of GIPC1 as transcription factor. The relevance of GIPC1 for tumor growth and metastasis was shown in mice intrasplenically transplanted with MACC1-overexpressing CRC cells, since GIPC1 knockdown reduced these MACC1-induced features. Very importantly, GIPC1 correlates with MACC1 expression in human primary CRC specimens, and is prognostic for metastasis formation and metastasis-free survival. Combination of MACC1 and GIPC1 expression improved patient survival prognosis, whereas SH3BP4 expression did not show any prognostic value.

For GIPC1, there are already more than 50 protein-protein interactions (PPI) described e.g. with transmembrane proteins (IGF1R, NTRK1, ADRB1, DRD2, TGFβR3, SDC4, SEMA4C, LRP1, NRP1, GLUT1, integrin α5 and VANGL2), or cytosolic signaling regulators (APPL1 and RGS19). This enables GIPC1 to be involved in a variety of crucial cellular and pathophysiological processes, loads PDZ ligands as cargoes for MYO6-dependent endosomal trafficking, and integrin recycling during cell migration, angiogenesis and cytokinesis (21, 26). These interactions are mainly enabled via its PDZ domain. For MACC1, several PPI are already described mainly identified by (phospho) mass spectrometry and verified by independent methods, e.g. for clathrin-mediated endocytosis-associated proteins such as CLTC, DNM2 and AP-2 (10), or for kinases, with 21 kinases belonging to the serine/threonine kinase family and for MEK1 (MAP2K1; dual specificity MAP kinase kinase 1, member of the very small family of dual specificity protein kinases phosphorylating substrates at tyrosine and subsequently at threonine residues) (35) with implications in MACC1-mediated cell proliferation and migration. The MACC1 domain structure is typical for signal transduction proteins, including for instance a SH3 (Src-homolgy 3) domain and a PXXP-motif (3, 16). Here we identified a different PPI mechanism of GIPC1 independent of the GIPC1 PDZ domain with a binding of the GIPC1 stretch aa 241-247 to MACC1 in conjunction with the MACC1 SH3 domain (aa 552-618).

However, the contribution to the same biological processes by both of the proteins, such clathrin-mediated endocytosis –for GIPC1 (37, 38) and for MACC1 (10)-, involvement to ERK activation –for GIPC1 (39) and for MACC1 (35)-, or impact on cellular migration –for GIPC (40–42) and for MACC1 (3, 4)- might underline a common regulation as well as functional interaction.

MACC1 expression is regulated in a variety of ways, e.g. by non-coding RNAs/miRNAs (4), and by transcription factors such as c-Jun, SP1 or C/EBP (36), thereby regulating transcriptional targets such as c-Met, Nanog, Spon2, ABCB1 or S100P (3, 8, 14, 43, 44). Here we describe for the first time GIPC1 as novel transcription factor, binding to the gene promoter of MACC1 (TSS to -60 bp). Stable knockdown of GIPC1 in a high MACC1 expressing CRC cell line resulted in a decrease in the MACC1 expression. Interestingly, the decrease in MACC1 levels was due to a decrease in the MACC1 promoter activity in GIPC1 knockdown cells compared with control cells. This indicated that GIPC1 governs a part of the MACC1 promoter activity. Thus, we carried out ChIP and EMSA experiments to depict the physical interaction of GIPC1 with the MACC1 promoter, and the results suggested, indeed, that GIPC1 binds to the MACC1 promoter and regulates its activity. This was the first study elucidating the role of GIPC1 as a transcription factor. Additionally, we demonstrated that GIPC1 binds to the first 60 bp upstream of the TSS, but it remains unclear whether GIPC1 acts as an accessory molecule to mediate transcription of the MACC1 gene or directly binds to the MACC1 promoter sequences and plays an essential role in oncogenic transformation mediated by MACC1.

We have previously shown that high MACC1 expression, either endogenous or by forced overexpression, induced tumor initiation, progression and metastasis in mice (3, 11, 13, 14). However, when diminishing GIPC1 expression in endogenously MACC1 high expressing cells, spleen tumor development and liver metastasis formation are reduced. Thus, targeting GIPC1 – gene-specifically by RNAi or by using short peptide– might represent a promising therapeutic approach [e.g. (30, 45)]. In addition, since first MACC1-targeting small molecules are meanwhile discovered (46, 47), combinatorial anti-tumoral/anti-metastatic intervention strategies targeting simultaneously GIPC1 and MACC1, might be beneficial.

For CRC patients, GIPC1 expression levels are of prognostic value for metastasis-free survival and correlate with MACC1 expression. Thus, GIPC1-targeting intervention strategies might interdict the MACC1-induced cancer metastasis formation. Combining both biomarkers (both low vs. both high) improved patient metastasis survival prognosis. In addition, another family member of GIPC1, GIPC2 – 62% aa homology with GIPC1, is also promoting metastasis formation of solid tumors. However, the only relative of MACC1, SH3PB4, although shown to interact with GIPC1, had no impact on MACC1-induced cell migration and invasion, and did not show any prognostic relevance in CRC patients.

In conclusions, we identified a novel, dual function of GIPC1 - as protein interaction partner and as transcription factor of MACC1 – for tumor progression and cancer metastasis. Clinical application of combined intervention strategies targeting MACC1 expression and GIPC1 expression might contribute to restrict MACC1-induced cancer metastasis and thus to improved patient survival.

## Data availability statement

The datasets presented in this study can be found in online repositories. The names of the repository/repositories and accession number(s) can be found in the article/**Supplementary Material**.

## Ethics statement

The studies involving humans were approved by Charité Ethics Committee, Charité University Medicine, Berlin, Germany. The studies were conducted in accordance with the local legislation and institutional requirements. The participants provided their written informed consent to participate in this study. The animal study was approved by Charité Ethics Committee, Charité University Medicine, Berlin, Germany. The study was conducted in accordance with the local legislation and institutional requirements.

## Author contributions

FS: Conceptualization, Investigation, Project administration, Writing – original draft, Writing – review & editing, Data curation, Formal analysis, Methodology. HS: Conceptualization, Data curation, Formal analysis, Investigation, Methodology, Project administration, Writing – original draft, Writing – review & editing, Funding acquisition. MJ: Data curation, Funding acquisition, Investigation, Writing – original draft, Writing – review & editing. JS: Data curation, Investigation, Writing – review & editing, Formal analysis. PH: Data curation, Formal analysis, Investigation, Writing – review & editing. DK: Data curation, Investigation, Writing – review & editing. KS: Data curation, Investigation, Writing – review & editing, Formal analysis. IF: Investigation, Writing – review & editing. WW: Investigation, Writing – review & editing. GD: Data curation, Formal analysis, Investigation, Writing – review & editing. RV: Data curation, Formal analysis, Investigation, Writing – review & editing. FR: Conceptualization, Funding acquisition, Investigation, Writing – review & editing. PS: Conceptualization, Funding acquisition, Investigation, Writing – review & editing. US: Project administration, Supervision, Writing – original draft, Investigation, Writing – review & editing, Conceptualization, Funding acquisition.
