# Supplementary material for: GIPC1 regulates MACC1-driven metastasis
Source: Front Oncol. 2023 Dec 8;13:1280977. doi: 10.3389/fonc.2023.1280977 (PMC10748395; doi:10.3389/fonc.2023.1280977)
Supplement: Supplementary file 1 [file DataSheet_1.pdf]

Yeast Two-Hybrid binding assay

Yeast strain: S. cerevisiae Y187

Interaction tested between: WT GIPC1 and WT SH3BP4 or WT MACC1

No.	BD-Plasmid	Amino acids	Blue colonies within			AD-Plasmid	Amino acids
			1 h	2h	4 h		
1	pGBT9[**GIPC1**]	1-333	-	-	-	pGAD10	-
2	pGBT9	-	-	-	-	pGAD10[r**SH3BP4**]	1-961
3	pGBT9[**GIPC1**]	1-333	+	+	+	pGAD10[r**SH3BP4**]	1-961
4	pGBT9	-	-	-	-	pGAD10[**MACC1**]	2-852
5	pGBT9[**GIPC1**]	1-333	+	+	+	pGAD10[**MACC1**]	2-852
6	pGBT9[r**SH3BP4**]	1-961	-	-	-	pGAD10	-
7	pGBT9	-	-	-	-	pGAD10[**GIPC1**]	1-333
8	pGBT9[r**SH3BP4**]	1-961	+	+	+	pGAD10[**GIPC1**]	1-333
9	pGBT9[**MACC1**]	2-852	-	-	-	pGAD10	-
10	pGBT9[**MACC1**]	2-852	+	+	+	pGAD10[**GIPC1**]	1-333

Interaction tested between: $GIPC1^{H191D}$ and WT SH3BP4 or WT MACC1

No.	BD-Plasmid	Amino acids	Blue colonies within			AD-Plasmid	Amino acids
			1 h	2h	4 h		
1	pGBT9[r**SH3BP4**]	1-961	-	-	-	pGAD10	-
2	pGBT9	-	-	-	-	pGAD424[**$GIPC1^{H191D}$**]	1-333
3	pGBT9[r**SH3BP4**]	1-961	+	+	+	pGAD424[**$GIPC1^{H191D}$**]	1-333
4	pGBT9[**MACC1**]	2-852	-	-	-	pGAD10	-
5	pGBT9[**MACC1**]	2-852	+	+	+	pGAD424[**$GIPC1^{H191D}$**]	1-333

Interaction tested between: $GIPC1^{98-333}$ and WT SH3BP4 or WT MACC1

No.	BD-Plasmid	Amino acids	Blue colonies within			AD-Plasmid	Amino acids
			1 h	2h	4 h		
1	pGBT9[**$GIPC1^{98-333}$**]	98-333	-	-	-	pGAD10	-
2	pGBT9	-	-	-	-	pGAD10[r**SH3BP4**]	1-961
3	pGBT9[**$GIPC1^{98-333}$**]	98-333	+	+	+	pGAD10[r**SH3BP4**]	1-961
4	pGBT9	-	-	-	-	pGAD10[**MACC1**]	2-852
5	pGBT9[**$GIPC1^{98-333}$**]	98-333	-	+	+	pGAD10[**MACC1**]	2-852
6	pGBT9[r**SH3BP4**]	1-961	-	-	-	pGAD10	-
7	pGBT9	-	-	-	-	pGAD10[**$GIPC1^{98-333}$**]	98-333
8	pGBT9[r**SH3BP4**]	1-961	+/-	+	+	pGAD10[**$GIPC1^{98-333}$**]	98-333
9	pGBT9[**MACC1**]	2-852	-	-	-	pGAD10	-
10	pGBT9[**MACC1**]	2-852	+/-	+	+	pGAD10[**$GIPC1^{98-333}$**]	98-333

Interaction tested between: GIPC1$^{1-134}$ and WT SH3BP4 or WT MACC1

No.	BD-Plasmid	Amino acids	Blue colonies within			AD-Plasmid	Amino acids
			1 h	2h	4 h		
1	pGBT9[**GIPC1$^{1-134}$**]	1-134	-	-	-	pGAD10	-
2	pGBT9	-	-	-	-	pGAD10[r**SH3BP4**]	1-961
3	pGBT9[**GIPC1$^{1-134}$**]	1-134	-	-	-	pGAD10[r**SH3BP4**]	1-961
4	pGBT9	-	-	-	-	pGAD10[**MACC1**]	2-852
5	pGBT9[**GIPC1$^{1-134}$**]	1-134	-	-	-	pGAD10[**MACC1**]	2-852
6	pGBT9[r**SH3BP4**]	1-961	-	-	-	pGAD10	-
7	pGBT9	-	-	-	-	pGAD10[**GIPC1$^{1-134}$**]	1-134
8	pGBT9[r**SH3BP4**]	1-961	-	-	-	pGAD10[**GIPC1$^{1-134}$**]	1-134
9	pGBT9[**MACC1**]	2-852	-	-	-	pGAD10	-
10	pGBT9[**MACC1**]	2-852	-	-	-	pGAD10[**GIPC1$^{1-134}$**]	1-134

Interaction tested between: GIPC1$^{98-134}$ and WT SH3BP4 or WT MACC1

No.	BD-Plasmid	Amino acids	Blue colonies within			AD-Plasmid	Amino acids
			1 h	2h	4 h		
1	pGBT9[**GIPC1$^{98-134}$**]	98-134	-	-	-	pGAD10	-
2	pGBT9	-	-	-	-	pGAD10[r**SH3BP4**]	1-961
3	pGBT9[**GIPC1$^{98-134}$**]	98-134	-	-	-	pGAD10[r**SH3BP4**]	1-961
4	pGBT9	-	-	-	-	pGAD10[**MACC1**]	2-852
5	pGBT9[**GIPC1$^{98-134}$**]	98-134	-	-	-	pGAD10[**MACC1**]	2-852
6	pGBT9[r**SH3BP4**]	1-961	-	-	-	pGAD10	-
7	pGBT9	-	-	-	-	pGAD10[**GIPC1$^{98-134}$**]	98-134
8	pGBT9[r**SH3BP4**]	1-961	-	-	-	pGAD10[**GIPC1$^{98-134}$**]	98-134
9	pGBT9[**MACC1**]	2-852	-	-	-	pGAD10	-
10	pGBT9[**MACC1**]	2-852	-	-	-	pGAD10[**GIPC1$^{98-134}$**]	98-134

Interaction tested between: GIPC1$^{123-227}$ and WT SH3BP4 or WT MACC1

No.	BD-Plasmid	Amino acids	Blue colonies within			AD-Plasmid	Amino acids
			1 h	2h	4 h		
1	pGBT9[**GIPC1$^{123-227}$**]	123-227	-	-	-	pGAD10	-
2	pGBT9	-	-	-	-	pGAD10[r**SH3BP4**]	1-961
3	pGBT9[**GIPC1$^{123-227}$**]	123-227	-	-	-	pGAD10[r**SH3BP4**]	1-961
4	pGBT9	-	-	-	-	pGAD10[**MACC1**]	2-852
5	pGBT9[**GIPC1$^{123-227}$**]	123-227	-	-	-	pGAD10[**MACC1**]	2-852
6	pGBT9[r**SH3BP4**]	1-961	-	-	-	pGAD10	-
7	pGBT9	-	-	-	-	pGAD10[**GIPC1$^{123-227}$**]	123-227
8	pGBT9[r**SH3BP4**]	1-961	-	-	-	pGAD10[**GIPC1$^{123-227}$**]	123-227
9	pGBT9[**MACC1**]	2-852	-	-	-	pGAD10	-
10	pGBT9[**MACC1**]	2-852	-	-	-	pGAD10[**GIPC1$^{123-227}$**]	123-227

Interaction tested between: GIPC1$^{222\text{-}333}$ and WT SH3BP4 or WT MACC1

No.	BD-Plasmid	Amino acids	Blue colonies within			AD-Plasmid	Amino acids
			1 h	2h	4 h		
1	pGBT9[**GIPC1$^{222\text{-}333}$**]	222-333	-	-	-	pGAD10	-
2	pGBT9	-	-	-	-	pGAD10[r**SH3BP4**]	1-961
3	pGBT9[**GIPC1$^{222\text{-}333}$**]	222-333	+	+	+	pGAD10[r**SH3BP4**]	1-961
4	pGBT9	-	-	-	-	pGAD10[**MACC1**]	2-852
5	pGBT9[**GIPC1$^{222\text{-}333}$**]	222-333	+	+	+	pGAD10[**MACC1**]	2-852
6	pGBT9[r**SH3BP4**]	1-961	-	-	-	pGAD10	-
7	pGBT9	-	-	-	-	pGAD10[**GIPC1$^{222\text{-}333}$**]	222-333
8	pGBT9[r**SH3BP4**]	1-961	+	+	+	pGAD10[**GIPC1$^{222\text{-}333}$**]	222-333
9	pGBT9[**MACC1**]	2-852	-	-	-	pGAD10	-
10	pGBT9[**MACC1**]	2-852	+	+	+	pGAD10[**GIPC1$^{222\text{-}333}$**]	222-333

Interaction tested between: WT GIPC2 and WT SH3BP4 or WT MACC1

No.	BD-Plasmid	Amino acids	Blue colonies within			AD-Plasmid	Amino acids
			1 h	2h	4 h		
1	pGBT9[**mGipc2**]	1-314	-	-	-	pGAD10	-
2	pGBT9	-	-	-	-	pGAD10[r**SH3BP4**]	1-961
3	pGBT9[**mGipc2**]	1-314	+	+	+	pGAD10[r**SH3BP4**]	1-961
4	pGBT9	-	-	-	-	pGAD10[**MACC1**]	2-852
	pGBT9[**mGipc2**]	1-314	-	+/-	+	pGAD10[**MACC1**]	2-852
5	pGBT9[**GIPC2**]	1-315	-	-	-	pGAD10	-
6	pGBT9[**GIPC2**]	1-315	-	-	-	pGAD10[r**SH3BP4**]	1-961
7	pGBT9[**GIPC2**]	1-315	-	-	-	pGAD10[**MACC1**]	2-852
8	pGBT9[r**SH3BP4**]	1-961	-	-	-	pGAD10	-
9	pGBT9	-	-	-	-	pGAD10[m**Gipc2**]	1-314
10	pGBT9[r**SH3BP4**]	1-961	+	+	+	pGAD10[m**Gipc2**]	1-314

11	pGBT9	-	-	-	-	pGAD10[**GIPC2**]	1-315
12	pGBT9[r**SH3BP4**]	1-961	-	-	-	pGAD10[**GIPC2**]	1-315
13	pGBT9[**MACC1**]	2-852	-	-	-	pGAD10	-
14	pGBT9[**MACC1**]	2-852	+	+	+	pGAD10[m**Gipc2**]	1-314
15	pGBT9[**MACC1**]	2-852	-	+/-	+	pGAD10[**GIPC2**]	1-315

Interaction tested between: WT GIPC3 and WT SH3BP4 or WT MACC1

No.	BD-Plasmid	Amino acids	Blue colonies within			AD-Plasmid	Amino acids
			1 h	2h	4 h		
1	pGBT9[**GIPC3**]	2-312	-	-	-	pGAD10	-
2	pGBT9	-	-	-	-	pGAD10[r**SH3BP4**]	1-961
3	pGBT9[**GIPC3**]	2-312	-	-	-	pGAD10[r**SH3BP4**]	1-961
4	pGBT9	-	-	-	-	pGAD10[**MACC1**]	2-852
5	pGBT9[**GIPC3**]	2-312	-	-	-	pGAD10[**MACC1**]	2-852
6	pGBT9[r**SH3BP4**]	1-961	-	-	-	pGAD10	-
7	pGBT9	-	-	-	-	pGAD10[**GIPC3**]	2-312
8	pGBT9[r**SH3BP4**]	1-961	-	-	-	pGAD10[**GIPC3**]	2-312
9	pGBT9[**MACC1**]	2-852	-	-	-	pGAD10	-
10	pGBT9[**MACC1**]	2-852	-	-	+	pGAD10[**GIPC3**]	2-312

Interaction tested between: MACC1^{P531A,P534A} and WT GIPC1

No.	BD-Plasmid	Amino acids	Blue colonies within			AD-Plasmid	Amino acids
			1 h	2h	4 h		
1	pGBT9[**MACC1**$^{\mathbf{P531A,P534A}}$]	2-852	-	-	-	pGAD10	-
2	pGBT9	-	-	-	-	pGAD10[**GIPC1**]	1-333
3	pGBT9[**MACC1**$^{\mathbf{P531A,P534A}}$]	2-852	-	-	+	pGAD10[**GIPC1**]	1-333
4	pGBT9[**GIPC1**]	1-333	-	-	-	pGAD10	-
5	pGBT9	-	-	-	-	pGAD10[**MACC1** $^{\mathbf{P531A,P534A}}$]	2-852
6	pGBT9[**GIPC1**]	1-333	+	+	+	pGAD10[**MACC1**$^{\mathbf{P531A,P534A}}$]	2-852

Interaction tested between: MACC1$^{\Delta SH3}$ and WT GIPC1

No.	BD-Plasmid	Amino acids	Blue colonies within			AD-Plasmid	Amino acids
			1 h	2h	4 h		
1	pGBT9[**MACC1$^{\Delta SH3}$**]	2-556;608-852	-	-	-	pGAD10	-
2	pGBT9	-	-	-	-	pGAD10[**GIPC1**]	1-333
3	pGBT9[**MACC1$^{\Delta SH3}$**]	2-556;608-852	-	-	-	pGAD10[**GIPC1**]	1-333
4	pGBT9[**GIPC1**]	1-333	-	-	-	pGAD10	-
5	pGBT9	-	-	-	-	pGAD10[**MACC1$^{\Delta SH3}$**]	2-556;608-852
6	pGBT9[**GIPC1**]	1-333	-	-	-	pGAD10[**MACC1$^{\Delta SH3}$**]	2-556;608-852

Supplementary Figure 1: Results of the β-galactosidase filter assay. To test for protein-protein interaction between variants of GIPC1, GIPC2, or GIPC3 and SH3BP4 or MACC1, respectively, the Y187 yeast reporter strain was co-transformed with the indicated combinations. Yeast colonies that could grow on SD agar plates without histidine, tryptophan, and leucine were lifted onto round filters. Yeast cells were then lysed in liquid nitrogen. The presence of yeast colonies that developed blue stain was recorded 1, 2, and 4 h after addition of the substrate X-Gal. Markings are as follows: "–" means that no blue colonies were visible; "+" that all colonies were stained blue; and "+/-" that only some of the colonies developed blue stain.
