# Supplementary material for: GIPC1 regulates MACC1-driven metastasis
Source: Front Oncol. 2023 Dec 8;13:1280977. doi: 10.3389/fonc.2023.1280977 (PMC10748395; doi:10.3389/fonc.2023.1280977)
Supplement: Supplementary file 2 [file DataSheet_2.docx]

**Supplementary Methods**

2.1 Cell lines, cell clones, RNAi

Human CRC cell lines SW48, SW480, SW620, LoVo, WIDR, DLD1, HCT116, and HCT15 were cultivated in Roswell Park Memorial Institute (RPMI)-1640 (PAA). HT29, HCA7, and Caco-2 were grown in Dulbecco’s modified Eagles’s Medium (DMEM) (Gibco), supplemented with 2 mM L-glutamine and 100 mg/l sodium pyruvate or 2mM L-glutamine and 1% NEAA, respectively. All cell lines were supplemented with 10% fetal bovine serum (FBS; Invitrogen).

Cells were incubated at 37°C in a humidified atmosphere containing 5% CO_2_. The cells were mycoplasma free and tested regularly. Authentication of the cell lines was performed by short tandem repeat (STR) genotyping. STR genotypes were consistent with published genotypes for these cell lines.

SW480 cells were stably transfected with the plasmids pcDNA3.1, pcDNA3.1/MACC1, pcDNA3.1/GIPC1 or pcDNA3.1/SH3BP4 by using Fugene HD (Roche) and treated twice a week with 1 mg/ml G418 (PAA), thereby generating SW480/vector, SW480/MACC1, SW480/GIPC1, and SW480/SH3BP4 cell clones. SW620 cells were stably transfected with the plasmids pSil2/control-shRNA or pSil2/GIPC1-shRNA (Thermo Fisher Scientific) generating the cell clones SW620/control-shRNA as well as SW620/GIPC1-shRNA. The transfectants were treated twice a week with 1 mg/ml G418 (PAA).

For RNAi experiments with siRNA, predesigned individual siRNA for MACC1, GIPC1, and SH3BP4 were obtained from Ambion. Scrambled siRNA from Ambion was used as negative control. For RNAi experiments, 3 × 10^5^ CRC cells were seeded in a 6 well plate. After 24 h, cells were transfected with 50 nM siRNA targeting MACC1, GIPC1, or SH3BP4 using RNAiMAX lipofectamine transfection reagent (Invitrogen) as per manufacturer’s protocol.

2.2 Plasmid constructs

Subcloning and polymerase chain reactions (PCR) were performed using standard methods. Full-length GIPC1 (*Homo sapiens*) was amplified by PCR from a HeLa cell cDNA library (Clontech) using primers (forward: 5'-tcttctggtgaccccacttc-3'; reverse: 5'-gtcatcatcgcagggtcc-3') and subsequently subcloned into the EcoRV site of pBlueScript-II-KS (+) (Agilent) to generate pBS[GIPC1]. pBS[GIPC1] served as a template for the PCR-amplification of DNA fragments encoding full-length or truncated variants of GIPC1 using the following primers [amino acid (aa) numbering refers to GenBank accession no. NP_005707]: GIPC1^aa1-333^ (forward: 5'-CCCGAATTCatgccgctgggactg-3'; reverse: 5'-CCCGAATTCctagtagcggccgac-3'), GIPC1^aa98-333^ (corresponding to isoform 2 of GIPC1 that lacks the first 97 aa of GIPC1 isoform 1) (forward: 5'-CCCGAATTCatgttctgcaccctgaac-3'; reverse: 5'-CCCGAATTCctagtagcggccgac-3'), GIPC1^aa1-134^ (forward: 5'-CCCGAATTCatgccgctgggactg-3'; reverse: 5'-CCCGAATTCctacacctccttgcgctg-3'), GIPC1^aa98-134^ (forward: 5'-CCCGAATTCatgttctgcaccctgaac-3'; reverse: 5'-CCCGAATTCctacacctccttgcgctg-3'), GIPC1^aa122-227^ (forward: 5'-CCCGAATTCatcttcgcccac-3'; reverse: 5'- CCCGAATTCctaacccgctgaacgctg-3'), GIPC1^aa222-333^ (forward: 5'-CCCGAATTCagccagcgttcag-3'; reverse: 5'-CCCGAATTCctagtagcggccgac-3'). The resulting PCR products were cleaved with the restriction enzyme EcoRI and inserted into the EcoRI-sites of vectors pGBT9 and pGAD10 (both Clontech), to generate fusion proteins of GIPC1 and the GAL4 DNA-binding domain or the GAL4 activation domain, respectively. Full-length cDNA clones for GIPC2 (clone IRATp970E1033D) and GIPC3 (clone IRCMp5012G0714D) were aquired from imaGenes (Germany) and served as templates for PCR-amplification of GIPC2 and -3 cDNAs. Full-length DNA fragments of 2 were sub-cloned into pGBT9 and pGAD10 (both Clontech) via a BamHI restriction site using the following primers: pGBT9[GIPC2] (forward: 5'-GGCGGATCCttatgcccctgaagctg-3'; reverse: 5'-GGCGGATCCtcataatcctcttcg-3') and pGAD10[GIPC2] (forward: 5'-GGCGGATCCatgcccctgaagctg-3'; reverse: 5'-GGCGGATCCtcataatcctcttcg-3'). Full-length DNA fragments of GIPC3 were sub-cloned into the EcoRI-restriction sites of pGBT9 and pGAD10 (both Clontech) using the following primers (forward: 5'-GGGGAATTCgagggagcagcggcc-3'; reverse: 5'-GGGGAATTCaaactagccacaggc-3'). Clones were verified by DNA sequencing.

A fusion protein of full-length Sh3bp4 fused in-frame to the Gal4 DNA-binding domain was produced by PCR amplification of the DNA encoding Sh3bp4 (*Rattus norvegicus*) from an adult rat brain cDNA library (Clontech) with BamHI sites added to the 5'- and 3'-ends, respectively, using specific primers: (forward: 5'-GGGGGATCCgtatggcagcccagcgcatc-3'; reverse: 5'-GGGGGATCCtcagatcacaaagtcatc-3'). The cleaved PCR product was inserted into the BamHI site of the pGBT9 vector.

For site-directed mutagenesis of Gipc1 at H191, the Transformer Site-Directed Mutagenesis kit (Clontech) was used to generate the H191D substitution within the PDZ domain of Gipc1. The corresponding C625G nucleotide substitution was introduced into the Gipc1 (*Rattus norvegicus*; Genbank accession no. NM_053341) encoding cDNA within the pSG5 expression vector (Stratagene) using the following oligonucleotides (mutagenic primer: 5'-ggctgtcggGattatgaggttgccag-3'; selection primer: 5'-ctggtcgactcCagactcttccg-3'). The modified Gipc1 cDNA was inserted in-frame into the pGAD424 vector (Clontech).

2.3 Generation of MACC1 promoter constructs

Based on our previous publication (1), we created a series of different 5’-truncated fragments of the -992 to -18 MACC1 promoter region inserted into the pGL4.17 vector using restriction enzymes (SacI, EcoRV, BglII), and resulting in pGL4.17/MACC1p_-426_, pGL4.17/MACC1p_-206_, and pGL4.17/MACC1p_-133_. All constructs were sequenced for the presence of the correct insert (Stratec Molecular).

2.4 RNA isolation and quantitative PCR

Cells (3 × 10^5^) were seeded in a 6 well plate and total RNA was isolated using the Roboklon kit according to manufacturer’s instructions (Genematrix). RNA was quantified (Nanodrop, Peqlab) and 50 ng RNA were reverse transcribed (20 μl reaction: 1 × RT Buffer, 1 U RNase inhibitor, 10 mM MgCl_2_, random hexamers, 250 μM pooled dNTPs and 2.5 U MuLV reverse transcriptase; all products from Applied Biosystems). The reaction was carried out for 15 min at 42°C, 5 min at 99°C and subsequent cooling at 4°C for 5 min (T300 thermocycler, Biometra).

Sequences of primers of probes used for qRT-PCR: MACC1 forward primer 5`-ttc ttt tga ttc ctc ccg tga-3’, reverse primer 5`-act ctg atg ggc atg tgc tg-3’, FITC-labeled probe 5`-gca gac ttc ctc aag aaa ttc tgg aag atc ta-3’, LCRed640- labeled probe 5`-agt gtt tca gaa ctt ctg gac att tta gac ga-3’, amplicon 136 bp; GIPC1 forward primer 5’-gca tcc tcc gac ttg aac ac-3’, reverse primer 5’-tgc cga ggt gat gtt ctg c-3’, FITC-labeled probe 5’-gtc ctc cag ccc gat ctg-3’, LCRed-labeled probe 5’-ccc agg agc ttg tcc atg tcc act t-3’, amplicon 144 bp; SH3BP4 forward primer 5’-aca aag tac ccg act ttc cag-3’, reverse primer 5’-cct ggt agt agc cga tgt ac-3’, FITC-labeled probe 5’-ctc-aag act gtg cgg cag aa-3’, LCRed-labeled probe 5’-aga acc act acc tgc tgg agt aca aga-3’, amplicon 199 bp; (synthesis of primers: BioTez; synthesis of probes: TibMolbiol and Sigma). G6PDH was used as housekeeping gene (Roche).

The cDNA was amplified using SYBR Green dye chemistry using the light cycler 480 (Roche Diagnostics) and the following PCR conditions: 95°C for 2 min followed by 45 cycles of 95°C for 7 s, 60°C for 10 s and 72°C for 20 s. The same protocol for cDNA synthesis and PCR has been employed for RNA from tumor samples. Data analysis was performed with LightCycler 480 Software release 1.5.0 SP3 (Roche). Mean values were calculated from duplicate qRT-PCR reactions. Experiments were carried out three independent times.

2.5 Luciferase promoter activity assay

Transfections were carried out in 24 well culture plates using Fugene HD (Roche) according to manufacturer’s instructions. Briefly, 7.5 × 10^3^ cells were plated per well and transfected with 1 µg of pGL4.17 vector with a MACC1 promoter fragment or pGL4.17 empty vector along with 100 ng of Renilla luciferase vector. DNA to lipid ratio of 1:3 was used for all the experiments. 24 h after transfection, luciferase activity was measured according to manufacturer’s protocol using dual luciferase reporter assay kit (Promega) with a luminometer (Tecan infinite 200 PRO).

2.6 Chromatin Immunoprecipitation assay (ChIP)

ChIP assay was performed using EZ ChIPTM kit from Millipore as per manufacturer’s instruction. All the reagents were provided in the kit unless stated. Cells (2 × 10^6^) were plated in 10 cm dishes. After 24 h, the cells were cross-linked with 1% formaldehyde for 10 min at room temperature, lysed and sonicated to release chromatin. Cell lysates were sonicated for 25 pulses at 40% output and centrifuged at 10,000 rpm for 10 min. Supernatant was collected in a new tube and diluted using dilution buffer and protease inhibitor. 1% of this solution was stored at 4°C until the elution step to be served as input control. The protein-DNA complexes were precipitated on addition of a polyclonal GIPC1 antibody to the chromatin solution obtained above, overnight at 4°C. Protein G beads were then added and incubated for another 2 h at 4°C.Non-bound protein was washed away twice with the wash buffers provided in the kit. The protein-DNA-complex was eluted from the beads with the elution buffer followed by centrifugation at 3,000 rpm for 1 min. Cross-linking of protein and DNA was reversed at 68°C overnight and residual protein was digested by proteinase K at 55°C for 2 h. DNA was purified by column purification. The extracted DNA was subjected to PCR (28 cycles at 94°C for 30 s and 60°C for 30 s and 72°C for 1 min) with MACC1 promoter primers. The PCR product was run on an agarose gel to visualize the precipitation of the MACC1 promoter. GAPDH was used as an unrelated gene to validate the specificity of binding observed.

2.7 Nuclear extract preparation and Electrophoretic mobility shift assay (EMSA)

EMSA was performed using the LightShift Chemiluminescent EMSA Kit as per manufacturer’s instruction (Thermo Scientific). Briefly, 2 × 10^6^ cells were seeded in a 10 cm culture dish and incubated for 24 h for adhering to the surface. Nuclear extracts were prepared using NE-PER nuclear and cytoplasmic extraction reagent as per manufacturer’s instruction (Thermo Scientific). 5’ labeled biotin oligonucleotides for the putative binding sites for GIPC1 were synthesized (Biotez) and annealed. Double stranded biotin-labeled oligonucleotides were incubated with 10 µl of nuclear extract for 30 minutes at room temperature. The remaining steps follow the LightShift Chemiluminescent EMSA Kit protocol. For the super shift assay, the anti-GIPC1 antibody was added before addition of the specific oligonucleotide and incubated for 30 minutes on ice, whereas 100-fold molar excess of unlabeled oligonucleotides was used in the competition experiments.

2.8 Protein Extraction, Western blotting, Immunofluorescence, co-Immunoprecipitation

These methods were performed as previously described (2). Briefly, after total protein extraction, cells were lysed in RIPA buffer and centrifuged for 10 min at 14,000 rpm. Lysates were separated on NuPage 10% Bis-Tris gels (Life Technologies) and transferred to Hybond C Extra nitrocellulose membranes (GE Healthcare). Membranes were incubated in blocking solution (5% nonfat dry milk, 1% bovine serum albumin) for 1 h at room temperature.

Western blotting for V5-tagged MACC1 protein was performed with a direct HRP-labeled monoclonal mouse anti-human V5-specific antibody (Invitrogen, 1:2,500, overnight, 4°C). For GIPC1 we used an antibody from ABCAM (ab 5951, 0.1 µg/ml), for SH3BP4 we used a polyclonal antibody from rabbit (0.75 µg/ml), and for β-tubulin serving as loading control with a monoclonal mouse anti-human β-tubulin antibody (BD, 1:1,000, overnight, 4°C). Antibody-protein-complexes were visualized with ECL reagent and exposure to CL-XPosure™ Films (Pierce).

For immunofluorescent staining, SW620 cells cultivated on PDL-coated 8-well multitest slides (MP Biomedicals) were fixed for 10 minutes with ice-cold 100% methanol and then washed twice with 1x PBS. After 60 minutes incubation in blocking buffer (1x PBS containg 5% normal goat serum and 0.3% Triton X-100), SW620 cells were incubated sequentially with goat polyclonal antiserum to GIPC1 (Abcam, ab5951), Alexa488-coupled donkey-anti-goat secondary antibody (Dianova), rabbit polyclonal antiserum to MACC1 (Proteus Biosciences, 25-6792), and donkey-anti-rabbit-Cy3 secondary antibody (Dianova) including DAPI for the visualization of nuclei, all supplied in antibody dilution buffer (1x PBS with 1% BSA and 0.3% Triton X-100). The cells were then washed with 1x PBS and mounted with Mowiol (Calbiochem). Immunofluorescence was analyzed using an LSM-710 confocal system (Zeiss).

For detection of the physical binding of MACC1 and GIPC1 or SH3BP4 and GIPC1, 5 × 10^6^ SW480/MACC1-wt cells were lysed (20 mM Tris-HCl pH 7.5, 150 mM NaCl, 0.1% NP40, 1 mM EDTA, 1% triton-X-100, supplemented with protease inhibitor cocktail) for 15 min on ice, harvested, and centrifuged for 10 min at 14,000 rpm. The polyclonal rabbit anti-human MACC1 antibody (HPA020103, Sigma, 5 µg) or the GIPC1 antibody (ABCAM ab 5951, 5 µg) was added to the supernatant and incubated overnight at 4°C. Immunoprecipitation with an unrelated monoclonal mouse anti-human β-tubulin antibody (BD Bioscience, 5 µg, overnight, 4°C) served as negative control, immunoprecipitations with the GIPC1 or the SH3BP4 antibody served as positive controls. One approach without immunoprecipitation served as technical control.

2.9 Subcellular fractionation

Cytoplasmic, membrane and nuclear proteins were isolated from whole cell lysates of SW620 cells using the Qproteome cell compartment kit (Qiagen) according to the manufacturer’s instructions. Extracted protein fractions were precipitated 15 min on ice by addition of a 4-fold volume of acetone. The precipitates were centrifuged for 10 min at 12,000 x g and 4°C and the supernatant was discarded. The dried pellets were dissolved in 1% CHAPS in PBS containing protease inhibitors (5 mM pepstatin A, 5 mM leupeptin, 20 U/μl aprotinin, 100 mM phenylmethyl sulfonyl fluoride). Equal amounts of protein extracts, resolved by SDS-PAGE and transferred onto nitrocellulose membranes, were probed with antibodies against GIPC1 (#25-6792, 0,1 µg/ml, Proteus Biosciences Inc.), GAPDH (NB300-221SS, 1:5,000, Novus Biologicals), Clathrin heavy chain (#610499, 0.25 µg/ml, BD Transduction Laboratories) and Histon H3 (ab1791, 1:30,000, Abcam) followed by incubation with alkaline phosphatase (AP)-conjugated goat anti-rabbit IgG (#111-055-003, 1:4,000, Dianova) or AP-conjugated rabbit anti-mouse IgG (#315-055-003, 1:4,000, Dianova) secondary antibodies.

2.10 Yeast two-hybrid (Y2H) analysis

The Matchmaker I yeast two-hybrid system (Clontech) was used for the analysis of protein-protein interactions according to manufacturer’s instructions. The yeast reporter strain Y187 was co-transformed with combinations of pGBT9 and pGAD10 or pGAD424 plasmids and grown on SD agar plates devoid of histidine, tryptophan and leucine to select for co-transformants. Yeast colonies were lifted onto round filters (Whatman), lysed by immersion in liquid nitrogen and subsequently soaked in Z buffer (60 mM Na_2_HPO_4_, 40 mM NaH_2_PO_4_, 10 mM KCl, 1mM MgSO_4_, pH 7.0, 50 mM β-mercaptoethanol) at 30°C for the analysis of β-galactosidase activity with X-gal as a substrate (3). Depending on the time when blue colonies could be observed the results of the β-galactosidase filter assay were scored as follows: +++ ≤ 1hour; ++ ≤ 2 hours; + ≤ 4 hours and - when only white colonies were visible after 4 hours of incubation time. Experiments were done in triplicate and combinations with the empty vectors served as negative controls. The original results of the β-galactosidase staining of transformed yeast colonies are shown in Supplementary Figure 1.

2.11 Mass Spectrometry

Shot-gun proteomics-mass spectrometry were performed to determine the MACC1 interactome. Immunoprecipitations of SW620 cells were carried out with 2 polyclonal rabbit anti-human MACC1 antibodies (HPA020103, HPA020081, Sigma) independently 4 times, as described previously (2). Briefly, samples were eluted from the affinity beads, proteins were converted to peptides in a two-step digestion using endopeptidase LysC and trypsin, peptides were separated on a reversed-phase column, ionized on a Proxeon ion source and directly sprayed into the mass spectrometer (Q-Exactive, Themo). The recorded spectra were analyzed using the MaxQuant software package (Version, 1.2.2.5).

14.

2.12 Pepspot analysis: determining the MACC1 binding site on GIPC1

Peptide arrays displaying overlapping peptides (15mers with one amino acid shift) of the C-terminal GIPC1-sequence (position 222 -333) were SPOT-synthesized on a Whatman-50 cellulose membranes (Whatman) using an automatic SPOT-synthesizer (INTAVIS AG).

After washing a synthesized array with DMF, ethanol, and three times with TBS buffer for 10 min each the array was incubated for 3 h with blocking buffer. MACC1 binding was probed by incubation the array with a solution of DDK-tagged MACC1 (OriGene) at a concentration of 10 µg/ml in blocking buffer at room temperature overnight. After three times of washing with TBS buffer (10 min each) the array was incubated with a solution of a mouse 4C5 anti-DDK IgG antibody (OriGene) in blocking buffer (1µg/ml) for 3 h at room temperature, then washed three times with TBS and finally treated with a solution of a horseradish-peroxidase conjugated anti-mouse IgG antibody (Sigma A-5906) in blocking buffer (1µg/ml)) at room temperature for 1.5 h followed by three times washing with TBS (10 min each). MACC1-binding was visualized by using a chemiluminescent substrate (Uptilight HRP, Uptima) and a Lumi-Imager (Roche). Further analysis of spot signal intensities was executed with the Genespotter software (MicroDiscovery GmbH). False positives spots could be excluded by probing arrays with antibodies alone (for practical details: 4,5).

2.13 Xenografting and in vivo experiments

All experiments were performed in accordance with the United Kingdom Coordinated Committee on Cancer Research (UKCCCR) guidelines. Briefly, 5 ×10^6^ SW620/control shRNA or SW620/GIPC1 shRNA cells in 30 µl PBS were intrasplenically transplanted into 6-8 week-old female NMRI:nu/nu mice (n=6 per group). At the ethical end point on day 40, the animals were sacrificed, spleens (site of transplantation) and livers (site of distant metastasis) were removed, documented, and liver metastases with a size larger than 1 mm were counted.

2.14 Patients and tissues

We analyzed tissue specimens from 59 patients suffering from CRC with informed written consent (approved by Charité Ethics Committee, Charité University Medicine, Berlin, Germany) as used in our previous study (6). All patients did not have a history of familial colon cancer and did not suffer from a second tumor of the same or a different entity. All patients were staged I, II, or III (not distantly metastasized at the time point of surgery). They were previously untreated, and patients’ tumors were surgically resected R0 (complete resection with no microscopic residual tumor). 23 patients formed distant metastases metachronously (after surgery), whereas 36 patients remained free of metastases. The follow-up data of all patients was documented for more than 5 years and up to 14 years (with a median follow-up of 6.3 years) after diagnosis. Metastasis-free survival was calculated from the date of histopathological diagnosis until the date when the development of distant metastases was observed. A comprehensive description of the analyzed tissues can be found in [Stein et al. 2009](#_ENREF_36) (6).

Tumor specimens (all were adenocarcinomas) were snap frozen in liquid nitrogen. For subsequent mRNA expression analyses, RNA was extracted by using TRIzol reagent (Invitrogen) including a DNase step. RNA quality was proven (2100 Bioanalyzer, Agilent), and concentration was measured (RiboGreen RNA Quantitation Kit, Invitrogen).

2.15 Statistics

The comparisons of two groups were done by t-tests. More than two groups were compared using ANOVA and appropriate post tests. Correlation between MACC1 and GIPC1 in cell lines and patient samples were evaluated by using Pearson and Spearman-rho test. All tests were two-sided, and P values ≤0.05 were considered to be statistically significant. In patient samples, the cut-off expression values were determined by receiver operating characteristic (ROC) analysis. The Kaplan–Meier method was used to estimate cumulative survival rates, and differences in survival rates were assessed using the log-rank test. P≤0.05 was considered statistically significant.

**References**

1. Juneja M, Ilm K, Schlag PM, Stein U. (2013) [Promoter identification and transcriptional regulation of the metastasis gene MACC1 in colorectal cancer.](https://pubmed.ncbi.nlm.nih.gov/23800415/) Mol Oncol. 7:929-43.

2. Kobelt D, Perez-Hernandez D, Fleuter C, Dahlmann M, Zincke F, Smith J, Migotti R, Popp O, Burock S, Walther W, Dittmar G, Mertins P, Stein U. (2021) [The newly identified MEK1 tyrosine phosphorylation target MACC1 is druggable by approved MEK1 inhibitors to restrict colorectal cancer metastasis.](https://pubmed.ncbi.nlm.nih.gov/34247190/) Oncogene. 40:5286-5301.

3. Schneider S, Buchert M, Hovens CM. (1996) [An in vitro assay of beta-galactosidase from yeast.](https://pubmed.ncbi.nlm.nih.gov/8780862/) Biotechniques. 20:960-962.

4. Buey RM, Sen I, Kortt O, Mohan R, Gfeller D, Veprintsev D, Kretzschmar I, Scheuermann J, Neri D, Zoete V, Michielin O, de Pereda JM, Akhmanova A, Volkmer R, Steinmetz MO, (2012) [Sequence determinants of a microtubule tip localization signal (MtLS).](https://pubmed.ncbi.nlm.nih.gov/22696216/) J Biol Chem. 287:28227-28242.

5. Volkmer R. (2009) Synthesis and application of peptide arrays: quo vadis SPOT technology. ChemBiochem **10**:1431-1442.

6. Stein U, Walther W, Arlt F, Schwabe H, Smith J, Fichtner I, Birchmeier W, Schlag PM. (2009) [MACC1, a newly identified key regulator of HGF-MET signaling, predicts colon cancer metastasis.](https://pubmed.ncbi.nlm.nih.gov/19098908/) Nat Med. 15:59-67.
